# Infrared heating as an adjunct to achieve vehicle occupant thermal comfort

**DOI:** 10.1186/2046-7648-4-S1-A82

**Published:** 2015-09-14

**Authors:** David Collins, Ramona Rednic, C Douglas Thake

**Affiliations:** 1 Department of Applied Science and Health, University of Coventry, Coventry, UK; 2 Jaguar Land Rover Research, University of Warwick, Coventry, UK

## Introduction

Traditionally vehicle occupants are warmed or cooled indirectly via heating, ventilation and air conditioning (HVAC) systems. The need to enhance electric vehicle range has fuelled interest in developing alternative, more energy efficient modes or adjuncts to HVAC systems to achieve thermal comfort. Infrared (IR) radiant heat, that falls directly on the individual, could be a more effective and energy efficient adjunct to traditional air heating methods. Therefore this investigation examined the impact of applying an IR heat source on vehicle occupants achieving thermal comfort.

## Methods

Eight thermoneutral preconditioned male participants, aged 27(8) years; height 1.76(0.05) meters; mass 76.5(11.2) kg, were exposed to a dynamic "pull up" from 5 to 25 °C (1 °C.2 min^-1^) in an environmental chamber on three occasions whilst sat on a car seat. A covered infrared heating panel (≈0.55 m^2 ^consuming 48 W electrical power; Qpunkt, Germany) was located in either the sun visor position (IRV); foot well position (in front of the lower leg; IRL); or absent (NoIR). Thermal comfort and sensation [1], desirability (bespoke 7-point scale developed from a 3-point scale [2]) were sought at 2 min intervals alongside participants marking their 'acceptability of the total thermal environment' on a visual analogue scale [3]. Rectal, aural and various skin surface temperatures were recorded.

## Results

Whole body thermal comfort improved alongside increased thermal sensation in all trials (P < 0.001) with enhanced thermal comfort and sensation (warmth) in IRV (P < 0.001; <0.01) and IRL (P < 0.05; <0.001) compared to NoIR. Acceptability of the total thermal environment reached 'just acceptable' after 10-12 min with IR heating (air temperature, T_air_, IRV, 7.6 - 8.4 °C; IRL, 7.3 - 8.2 °C) with this point being reached at 16 min (T_air _9.9 °C) in NoIR (vs. IRV, P < 0.001; vs. IRL, P < 0.05). Likewise T_air _desirability was lower (a greater rise in temperature being desirable) in NoIR vs. IRV (P < 0.001) and IRL (P < 0.01) up to ≈24-28 min (T_air_, NoIR, 14.6 - 17.2 °C; IRV, 14.7 - 17.2 °C; IRL 15.1 - 17.7 °C). Head, face and nose thermal comfort and sensation did not vary between conditions. IR panel position dependent differences were evident for thermal comfort and sensation, respectively, for the chest & arms (P = 0.01; <0.05) and hands (P < 0.001) in IRV and the lower leg (P < 0.001), feet (p < 0.05; <0.01) and toes (P = 0.05; = 0.001) in IRL compared to NoIR. Both IRV and IRL resulted in improved thermal comfort and sensation of body areas in contact with the seat surface compared to NoIR; aligned to lower seat temperature desirability in NoIR compared to IRV (P < 0.01) and IRL (P < 0.05).

## Discussion

Incident IR radiation from the heating panel in IRV and IRL, provided heat energy that temporally enhanced local and overall thermal sensation and comfort compared to NoIR. The lack of difference in head, face and nose thermal comfort and sensation in IRV is likely due to the IR panel position limiting incident IR to these body segments. However the chest & arms and hands did benefit. IRL improved feet and toe thermal comfort and sensation, body areas that are notoriously cool and resistant to warming.

## Conclusion

The addition of an IR heating panel during a dynamic "pull up" protocol yielded positive and position specific effects on thermal perceptions in young healthy males. Therefore studies to further define the application of IR heating panels within vehicle cabins are warranted.
